# Low vapour pressure deficit affects nitrogen nutrition and foliar metabolites in silver birch

**DOI:** 10.1093/jxb/erw218

**Published:** 2016-06-03

**Authors:** Jenna Lihavainen, Viivi Ahonen, Sarita Keski-Saari, Sari Kontunen-Soppela, Elina Oksanen, Markku Keinänen

**Affiliations:** ^1^University of Eastern Finland, Department of Environmental and Biological Sciences, PO Box 111, 80101 Joensuu, Finland; ^2^University of Eastern Finland, Department of Environmental and Biological Sciences, PO Box 1627, 70211 Kuopio, Finland

**Keywords:** Air humidity, amino acids, *Betula*, *pendula*, carbohydrates, flavonoids, metabolite profiling, mineral nutrients, nitrogen, starch, VPD.

## Abstract

Air humidity indicated as vapour pressure deficit (VPD) is directly related to transpiration and stomatal function of plants. We studied the effects of VPD and nitrogen (N) supply on leaf metabolites, plant growth, and mineral nutrition with young micropropagated silver birches (*Betula pendula* Roth.) in a growth chamber experiment. Plants that were grown under low VPD for 26 d had higher biomass, larger stem diameter, more leaves, fewer fallen leaves, and larger total leaf area than plants that were grown under high VPD. Initially, low VPD increased height growth rate and stomatal conductance; however, the effect was transient and the differences between low and high VPD plants became smaller with time. Metabolic adjustment to low VPD reflected N deficiency. The concentrations of N, iron, chlorophyll, amino acids, and soluble carbohydrates were lower and the levels of starch, quercetin glycosides, and raffinose were higher in the leaves that had developed under low VPD compared with high VPD. Additional N supply did not fully overcome the negative effect of low VPD on nutrient status but it diminished the effects of low VPD on leaf metabolism. Thus, with high N supply, the glutamine to glutamate ratio and starch production under low VPD became comparable with the levels under high VPD. The present study demonstrates that low VPD affects carbon and nutrient homeostasis and modifies N allocation of plants.

## Highlight

Changing leaf to air vapour pressure deficit (VPD) modifies primary metabolism of plants as demonstrated by excess starch synthesis and reduced amino acid, chlorophyll, and nitrogen concentrations in leaves under low VPD.

## Introduction

Relative air humidity (RH) is associated with precipitation and cloudiness, which will increase in the northern latitudes together with temperature, atmospheric water vapour content, and frequency of wet days (Kont *et al.*, 2003; IPCC, 2013). In association with this development, RH is expected to increase in northern Europe. Air humidity is an important factor influencing plant growth and nutritional status. Water evaporation is driven by the difference of vapour pressure deficit (VPD) between leaf tissues and the atmosphere; therefore, low VPD decreases the evaporative demand and steady-state transpiration rate of plants (Adams and Hand, 1993; Yang *et al.*, 2012). Low VPD promotes stomatal opening, facilitating carbon dioxide (CO_2_) influx to the leaf mesophyll (Bunce, 1984; Gisleröd and Nelson, 1989). As a consequence, under low VPD, plants can assimilate CO_2_ at relatively small cost as regards water, but at the same time the decreased transpiration and mass flow of water may impair the mineral nutrient uptake of plants (Barber, 1962; Barber *et al.*, 1963; Cramer *et al.*, 2009).

McDonald *et al.* (2002) showed that exposure to low VPD or elevated CO_2_ decreased transpiration and ^15^N uptake in cottonwood (*Populus deltoides* Bartr). Although N uptake to the roots is achieved by specialized transporters for ammonium (NH_4_^+^) (ammonium transporter, AMT) or nitrate (NO_3_^−^) (nitrate transporter, NRT) (Crawford and Glass, 1998; Gojon *et al.*, 2011; Xu *et al.*, 2012), the transpiration rate affects uptake of water by the roots and thus the amount of nutrients available for absorption by the roots (Barber, 1962). Besides N, uptake of other mineral nutrients, such as phosphorus (P), potassium (K), magnesium (Mg), calcium (Ca), iron (Fe), and sulphur (S) also benefits from a high transpiration rate (Novak and Vidovic, 2003; Cernusak *et al.*, 2009; Shimono and Bunce, 2009; Shrestha *et al.*, 2015). In the free air humidity manipulation (FAHM) field experiment, a long-term exposure to elevated air humidity reduced growth rate, sap flux density, and leaf N and P content of silver birch and hybrid aspen trees (Kupper *et al.*, 2011). There is evidence that nutrient availability in the soil affects the transpiration rate of plants (Cramer *et al.*, 2008; Matimati *et al.*, 2014).

N, and nitrate in particular, regulates the genes that contribute to N assimilation, nitrate transport, and the production of organic acids, which act as counter anions for NO_3_^−^ and prevent cellular alkalinization (Wang *et al.*, 2000; Scheible *et al.*, 2004). Nitrate reductase (NIA) and nitrite reductase (NiR) reduce nitrate to nitrite and nitrite to ammonium, respectively. Ammonium is then fixed by the glutamine synthetase/glutamate synthase (GS/GOGAT) pathway to glutamine and glutamate, which are precursors for other amino acids. The tricarboxylic acid (TCA) cycle, in turn, provides carbon skeletons for the biosynthesis of amino acids. Thus, carbon and N metabolisms are tightly integrated processes, and plants need to balance their carbon fixation according to the N assimilation rate (reviewed in Stitt and Krapp, 1999).

N-limited plants often accumulate carbohydrates (Paul and Driscoll, 1997), which in turn inhibit photosynthesis (Paul and Foyer, 2001). N-limited plants store carbon as starch (Paul and Driscoll, 1997; Scheible *et al.*, 1997), which serves as a transient sink for excess photosynthates when sucrose synthesis and export is restricted (Paul and Foyer, 2001). Carbon-based secondary compounds produced via the phenylpropanoid pathway typically increase (Scheible *et al.*, 2004; Urbanczyk-Wochniak and Fernie, 2005), whereas the content of soluble carbohydrates may decrease under N deficiency (Geiger *et al.* 1999).

Plant growth can be inhibited under elevated air humidity as nutrients become limited (Tullus *et al.*, 2012; Sellin *et al.*, 2013). On the other hand, low VPD can enhance plant growth, due to ameliorated water status and carbon fixation (Gisleröd and Nelson, 1989; Schüssler *et al.*, 1992; Mortensen, 2000; Fordham *et al.*, 2001; Leuschner, 2002). In a field experiment, birch trees acclimated to low VPD by increasing the formation of fine roots and root tips, and specific leaf area (SLA) (Parts *et al.*, 2013; Sellin *et al.*, 2013; Rosenvald *et al.*, 2014). Ameliorated water status by low VPD can enhance turgor in leaves, which in turn induces cell expansion, cell division, and thus leaf area (Codarin *et al.*, 2006; Lendzion and Leuschner, 2008; Carins Murphy *et al.*, 2013).

We conducted a controlled growth chamber experiment with young micropropagated silver birch (*Betula pendula* Roth.) to study VPD effects on leaf metabolism as affected by N availability. As far as we know, this is the first study focusing on the metabolic adjustments of primary carbohydrate and N metabolism to altered water, carbon, and nutrient supply under low VPD. Since changes in the leaf metabolome and transcript levels are the basis of phenotypic responses to environmental factors, we studied plant growth, leaf traits (leaf area, SLA, water content, stomata, leaf veins), mineral nutrient content, N-related gene expression patterns (NIA, NiR, and NRT), and leaf metabolites as affected by VPD and N availability.

Low VPD may reduce the acquisition and translocation of N via transpiration flux and promote carbon fixation by stomatal opening. Any change in water, carbon, and nutrient resources would require readjustment of primary carbohydrate and N metabolism that was studied by metabolite profiling of leaves developed under different VPD levels. We hypothesized that under low VPD (i) plants would have a lower N concentration and lower levels of foliar N-containing metabolites (chlorophyll and amino acids) and higher levels of starch than under high VPD; (ii) the foliar expression levels of NIA, NiR, and NRT genes would be repressed under low VPD; but (iii) high N supply would induce the expression of N-related genes and ameliorate the negative effects of low VPD on N status.

## Materials and methods

### Experimental design

Silver birch (*Betula pendula* Roth.) genotype V14 from Vehmersalmi (62°N, 28°E, eastern Finland) was micropropagated by *in vitro* tissue culture. Six-month-old clonal birches (height ~40cm) were planted and grown in fertilized peat (10:8:16 N:P:K, Kekkilä) and vermiculite (1:1, v/v) in plastic pots (1.6 litres). The birches were transferred from the greenhouse to controlled growth chambers to acclimate for 4 d before the start of the treatments. In the chambers, the photoperiod and temperatures were adjusted to be similar to the greenhouse conditions and typical for summer at the latitude of Finland, 22:2h light:dark, 23 °C and 16 °C during light and dark periods, respectively. Towards the light/dark period, half of the lamps were switched on/off followed by the remaining lamps after 1h. With all the lamps on, the light intensity was 450 µmol m^−2^ s^−1^ at the crown level.

There were two humidity levels, 60% RH (high VPD, similar to greenhouse conditions) and 95% RH (low VPD), with two replicate chambers for each. VPD was 1.12 kPa and 0.73 kPa in the high VPD treatment and 0.14 kPa and 0.09 kPa in the low VPD treatment during the light and dark periods, respectively. Since the water use of plants and water evaporation from the soil differed between the VPD treatments, the water status of the soil was monitored every day by weighing the pots. Water status was maintained above 50% of the maximum water-holding capacity of soil. Typically, in high VPD conditions, plants were watered four times per week, and in low VPD conditions three times per week. Based on the pot masses, the amount of water required to reach field capacity was estimated, and added to the pots.

The plants were fertilized with 0.2% nutrient solution (19:5:20 N:P:K including micronutrients, Superex-9, Kekkilä, Finland) once a week (15mg of N). In each chamber, half of the plants were given an additional 15mg of N fertilization (+N) once a week as 0.08% ammonium nitrate solution (NH_4_NO_3_; JT Baker). Fertilization levels corresponded to N addition of 35kg^−1^ ha^−1^ and 70kg^−1^ ha^−1^, and total N supply (pre-mixed and added fertilization) was 112mg and 172mg of N, at the lower and higher fertilization levels, respectively. Altogether, 80 birches were divided randomly into the four chambers. There were 20 plants in each of the four treatments: high VPD, high VPD+N, low VPD, and low VPD+N. Plant positions were changed frequently inside the chambers and once between the two replicate chambers to diminish the possible chamber effects.

### Growth and biomass

Stem height was measured every third day, and stem diameter (5cm above ground) once a week. The height to diameter ratio (HDR) was calculated on the 24th day of the experiment. Stem height growth rate was calculated as height increment per day. Relative height growth (RHG %) and diameter growth (RDG %) were calculated based on the initial measurement (day 0) and the final measurement, which was done on day 26 for height and day 24 for diameter.

After harvesting plants and washing the roots, all plant parts were dried to constant weight at 45 °C for 3 d. The dried leaves, stems, and roots were weighed and the number of branches and leaves were calculated. Total biomass was calculated as the sum of leaf, stem, and root biomass. The mass fractions were obtained by dividing leaf, stem, or root dry mass by total biomass. The development of new branches and fallen leaves was determined by comparing photographs of plants taken on the fourth and 20th days of the experiment.

### Chlorophyll, stomatal response, and mineral nutrients

Chlorophyll content was measured as the chlorophyll content index (CCI) using a Chlorophyll Content Meter CCM200 (Opti-Sciences). Chlorophyll content was measured once a week from two marked leaves, which were fully expanded at the beginning of the experiment (old leaves). At the end of the experiment, chlorophyll content was measured from one new leaf developed during the experiment (fifth leaf from the top), which was then sampled for metabolite and gene expression analyses.

Stomatal conductance (*g*_s_, mmol m^−2^ s^−1^) was measured under high VPD (60% RH) once a week from the same two marked leaves, which were fully expanded at the beginning of the experiment (Decagon leaf porometer SC-1) (*n*=10).

The N concentration (mg g DW^−1^) was determined from new leaves and stem and supposed new roots developed during the experiment. Dried samples (leaf, stem, and roots) (~0.5g) from eight plants (*n*=8) per treatment were milled, and N concentration was determined by the Kjeldahl method. Total N content was estimated by multiplying the mass-based N concentration by biomass.

Concentrations of other mineral nutrients (mg g DW^−1^) (B, Ca, Co, Cr, Fe, K, Mg, Mn, Mo, Na, P, S, Sb, Se, V, and Zn) were determined from new leaves grown during the experiment, using an inductively coupled plasma optical emission spectrometer (ICP-OES, IRIS Intrepid II XSP). Dried leaves from six plants per treatment (*n*=6) were milled and leaf powder (~0.5g) was dried at 105 °C prior to the analysis. The samples were digested by the standard EPA-3051 method: microwave digestion (MARS5) with nitric acid and water (6:1 HNO_3_:H_2_O).

### Leaf traits

Leaf area (cm^2^) was determined using LAMINA software (Bylesjö *et al.*, 2008) from leaves grown during the experiment, one leaf per plant. Specific leaf area (SLA) was calculated as leaf area (cm^2^) divided by leaf dry weight (g DW^−1^) (*n*=20). Leaf water content (LWC) was determined after drying the leaves to constant weight (DW) at 50 °C for 2 d (*n*=20). LWC was then calculated as a function of (FW–DW) FW^−1^. Total leaf area was estimated as single leaf area multiplied by the number of leaves.

Anatomical traits of the leaf surface were studied with a light microscope from nail polish imprints, from a dry, pressed, fully expanded leaf grown during the experiment. Imprints were produced only for the lower side, as stomata are present only on the abaxial side of the leaf surface. Imprints were taken between the midribs at the widest part of the leaf. Three photographs of the imprint were taken at ×20 magnification for stomatal density measurement (number of stomata mm^−2^), and one photograph at ×5 magnification was taken for leaf vein density (vein length mm mm^−2^) measurement. The photographs were calibrated against a micrometer slide. Stomatal density and leaf vein density were calculated with ImageJ software (version 1.48). The total number of stomata and vein length per leaf were estimated by multiplying the results by the leaf area.

### Sampling and homogenization of metabolite and gene expression samples

Single leaf discs (ø 1cm) were taken from three fully expanded leaves (fourth, fifth, and sixth leaf from the top) grown during the experiment, and these three discs were pooled for metabolite analysis (~50mg FW). Two leaf discs were taken from the same three leaves, and six discs were pooled for gene expression analysis (~100mg FW). The samples were frozen in liquid nitrogen and stored at –70 °C.

Metabolite and gene expression samples were kept frozen while homogenized in 2ml safe-lock Eppendorf tubes with a bead mill (3×15s, 20 Hz, with a 5mm stainless steel bead) (TissueLyser, Qiagen, Germany).

### Metabolite extraction

Soluble metabolites were extracted from the leaf powder in two steps, first with 1ml of 100% methanol (Merck) and next with 1ml of 80% (v/v) aqueous methanol. Internal standard solution [benzoic-d_5_ acid 0.55mg ml^−1^ (Campro Scientific GmbH), glycerol-d_8_ 0.54mg ml^−1^ (Campro), 4-methylumbelliferone 2.29mg ml^−1^ (Sigma), dl-alanine-2,3,3,3-d_4_ 1.12mg ml^−1^ (Isotec)] in H_2_O:MeOH:DMSO (BDH Chemicals) (1:3:5) was added to each sample during the first extraction step. During both steps, the samples were extracted at 4 °C for 15min at 1400rpm (Thermomixer, Eppendorf, Germany) and centrifuged at 10 °C for 3min at 13 000rpm (13 500 *g*). The supernatants were then combined for metabolite analysis. In addition, quality control (QC) samples were produced by combining leaf extracts from samples of each treatment. The leaf extracts (100 µl) were dried in a vacuum; the vials were then degassed with N and stored at –70 °C overnight.

### Soluble carbohydrates and starch

The total soluble carbohydrate concentration of leaves (mg g FW^−1^) was determined by phenol–sulphuric acid assay, by mixing 50 µl of leaf extract with 200 µl of 5% phenol (JT Baker) solution in water (w/v) and 600 µl of sulphuric acid (Merck). The samples were heated at 95 °C for 5min, cooled to room temperature, and measured for absorbance at 490nm (Varian Cary 50, Agilent). Soluble carbohydrate concentration was determined using a standard curve of d-glucose (Sigma).

Starch (mg g FW^−1^) was determined after the extraction of soluble metabolites from the vacuum-dried plant residue (stored at –70 °C). The pellets were homogenized in 1ml of water with a bead mill (60s, 20 Hz, with a 5mm stainless steel bead). The homogenate was transferred to 10ml tubes and diluted to 5ml with water. The starch was gelatinized by heating 0.5ml of the homogenate at 100 °C for 60min at 200rpm. Gelatinization of starch was confirmed by light microscopy, by staining the precipitate with Lugol’s solution. The starch was digested to glucose with α-amyloglucosidase (from *Aspergillus niger*, Sigma) and α-amylase (from *Bacillus licheniformis*, Sigma). The reaction mixture contained 0.5ml of the sample and 0.5ml of enzyme solution, which included 6U of α-amyloglucosidase and 1U of α-amylase in a 200mM sodium acetate (JT Baker) buffer (pH 4.8). The samples were incubated at 50 °C for 20h. After incubation, enzyme activity was stopped by heating the samples at 100 °C for 2min. The samples were centrifuged at 10 °C for 5min at 13 000rpm (13 500 *g*), and glucose was determined from the supernatant by enzymatic assay with hexokinase and glucose 6-phosphate dehydrogenase (Glucose HK assay kit, Sigma).

### Metabolite analysis by gas chromatography–mass spectrometry

Metabolites were analysed from the leaf extracts of 10–12 plants from each treatment. A QC sample was included in the sample set on each day of GC-MS analysis. The frozen samples were allowed to reach room temperature before adding 20 µl of alkane series (C10–C40, Supelco) solution in hexane (Merck) as a retention time standard, and dried in a vacuum. The samples were redissolved in 40 µl of methoxyamine hydrochloride (MAHC, Sigma) (20mg ml^−1^) in pyridine (Sigma) and incubated for 180min at 30 °C at 200rpm. Finally, the samples were derivatized using 80 µl of *N*-methyl-*N*-(trimethylsilyl) trifluoroacetamide with 1% trimethylchlorosilane (MSTFA with 1% TMCS, Thermo Scientific) for 120min at 60 °C at 200rpm.

The GC-MS system consisted of the Agilent 6890N gas chromatograph system, mass spectrometer (5973N), autosampler (7683), and injector (7983) (Agilent Technologies, USA). Split injection (1 µl) was employed, using a deactivated Split precision liner (Restek) with a split ratio of 30:1. The column was 30 m Rxi-5Sil MS, 0.25mm ID, 0.25 µm df with 10 m Integra-Guard (Restek). The injection temperature was set to 260 °C, MSD interface 290 °C, MS source 230 °C, and MS quad 150 °C. Helium (1ml min^−1^) was used as a carrier gas. The oven temperature program was as follows: 3min isothermal heating at 70 °C, followed by a 6 °C min^−1^ ramp to 330 °C, 6min at 330 °C, and post-run at 70 °C for 3min. Mass spectra were collected with a scanning range of 55–550 *m/z* during 5.50–30min and 55–650 *m/z* during 30–49.50min.

Deconvolution, component detection, and quantification were conducted with Metabolite Detector (2.06 beta) (Hiller *et al.* 2009), and co-eluting components were confirmed by AMDIS (version 2.66) (NIST). The relative content of metabolite was calculated as metabolite peak area normalized by the peak area of the internal standard, benzoic-d_5_ acid, and the fresh weight of the sample.

Altogether, 169 compounds were detected from birch leaves by untargeted GC-MS analysis (see Supplementary Table S1 at *JXB* online). Relative standard deviation (RSD %) was calculated from QC samples (*n*=3), and it was 16.5% including all the 169 metabolites and 5.6% including only the internal standards. The metabolites were annotated by spectral data and retention time index matched to the NIST Mass Spectral Database (version 2.2, Agilent Technologies, National Institute of Standards and Technology, USA), the Golm Metabolome Database (GMD) (Schauer *et al.*, 2005; Hummel *et al.*, 2010), and standard compounds. Metabolite annotation details are described in Supplementary Table S1.

### RNA extraction and gene expression analysis by quantitative real-time PCR

RNA was isolated using the modified cetyltrimethylammonium bromide (CTAB) method (Chang *et al.*, 1993) and treated with DNase I. Total, DNA-free RNA was further purified using a Qiagen Plant RNA isolation kit. Concentration and purity were analysed using a NanoDrop spectrophotometer (Thermo Scientific). RNA was reverse-transcribed using a Transcriptor First Strand cDNA Synthesis Kit (Roche) and oligo(dT_18_) primer. Quantitative real-time PCRs were performed using 480 SYBR Green I Master Mix (Roche) in a 10 µl reaction volume with 0.5 µM gene-specific primers and 2.5 µl of diluted cDNA, deriving from 7.5ng of total RNA, as a template.

The quantitative PCR primers for *NIA* and *NiR* were designed using silver birch nitrate reductase (X54097) and nitrite reductase sequences (X60093), respectively. The primer for the nitrate transporter gene *NRT* was designed using a silver birch EST (Aalto and Palva, 2006) with a high homology to Arabidopsis nitrate transporter (AT3G21670). Primers for the reference gene, elongation factor 1α (*EF-1*α), were designed from an EST with homology to Arabidopsis *EF-1α* (AT1G07920). The primers used in this study are given in Supplementary Table S2. Tubulin primers are described in Ibrahim *et al.* (2010).

The reactions were performed in triplicate in a LightCycler 480 (Roche). The PCR conditions were as follows: 95 °C for 5min, followed by 40 cycles of 95 °C for 20s, 58 °C for 20s, and 72 °C for 20s. After the final extension (72 °C, 5min), a melt curve analysis was performed by lowering the temperature from 95 °C to 65 °C at 0.1 °C intervals. The fold change in gene expression was calculated using the comparative Ct method (2^−∆∆Ct^) (Livak and Schmittgen 2001), using *EF-1*α as a reference gene.

We tested EF-1α, actin (act), tubulin (tub), and ubiquitin (ubq) to be used as reference genes in quantitative real-time PCR. The expression of *EF-1*α and actin was the most stable in our system according to GeNorm (http://medgen.ugent.be/~jvdesomp/genorm/). However, as the expression of actin fluctuated in some samples, we used the *EF-1α* gene as an internal standard to normalize the quantification of mRNA expression.

The melting curve of the reference and target genes showed a single peak, indicating that a single PCR product was present in each reaction. PCR efficiencies were measured for each primer pair used, and were generally between 1.942 (*NIA*) and 2.031 (*EF-1α*), with slopes ranging from –3.25 to –3.47.

### Pattern recognition and statistics

For metabolite data, we used log10-transformed values without missing value imputation. The main effects of VPD and N and their interaction were tested using two-way ANOVA (IBM SPSS Statistics 19). False discovery rate (FDR) correction for multiple analysis was conducted for the individual GC-MS metabolites, *P*-values for each effect and interaction were treated separately, and a *q*-value <0.05 was considered significant (Benjamini and Hochberg, 1995) (Supplementary Table S1). Principal component analysis (PCA) was performed with Simca P+ (12.0.1, Umetrics, Umeå, Sweden) to observe general patterns and variation in the GC-MS metabolite data set (Pareto-scaled).

The initial measurements for height and diameter as well as the number of branches were used as covariates, when testing the treatment effects on those parameters (two-way ANOVA). Graphical vector analysis (GVA) was constructed to inspect whether the change in compound/nutrient concentration is due to altered uptake/biosynthesis or due to biomass dilution. The construction of GVA is described in Haase and Rose (1995), and the use of GVA to interpret changes in compound levels is discussed in Koricheva (1999). In GVA, high VPD treatment was considered as the comparison point, since the growth conditions were similar to the greenhouse conditions. Relationships between phenotypic parameters, mineral nutrient concentrations, transcript levels, and metabolites were inspected by means of correlation analysis (Pearson correlation, SPSS).

## Results

### Growth and biomass allocation

**Fig. 1.**
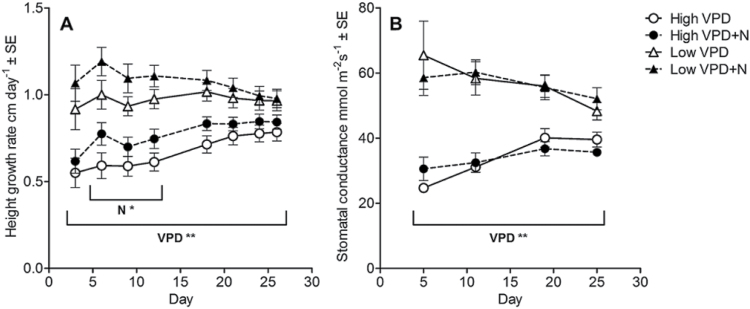
Stem height growth rate (A) and stomatal conductance (B) as affected by VPD and N treatments. Stomatal conductance was measured in high VPD conditions (60% RH) from two marked leaves that were fully expanded before the start of the treatments (old leaves). Data are presented as mean ±SE, *n*=20 for height growth and *n*=10 for stomatal conductance; two-way ANOVA **P<0.01, *P<0.05.

Plants showed higher relative height growth (RHG %) and diameter growth (RDG %) in low VPD than in high VPD (Table 1). Diameter growth increased more than height growth under low VPD compared with high VPD (Table 1). As a result, the HDR was lower in low VPD than in high VPD after 24 d (Table 1). At first, low VPD treatment increased the height growth rate (Fig. 1A). However, the stem height growth rate accelerated under high VPD, while the growth rate was constant (low VPD) or slowed down (low VPD+N) under low VPD (Fig. 1A) as the experiment progressed. Additional N fertilization tended to increase the height growth rate (Fig. 1A).

**Table 1.** Growth, biomass, leaf traits, and total N content as affected by VPD and N treatments Relative stem height growth, biomass, leaf traits, and N content are measured at day 26, relative stem diameter growth and height to diameter ratio at day 24, and the number of fallen leaves between day 4 and day 20.

	*n*	High VDP	High VPD+N	Low VPD	Low VPD+N	Effect		
						VDP	N	VDP×N
**Relative stem height growth (%**)	20	24.9±1.3	28.6±1.4	33.4±2.1	33.9±1.4	**	NS	NS
**Relative stem diameter growth (%**)	20	18.5±2.2	21.2±2.4	31.1±2.9	31.4±2.4	**	NS	NS
**Height to diameter ratio**	20	12.9±0.5	12.4±0.4	11.7±0.4	11.6±0.4	*	NS	NS
**Total biomass (g DW**)	8	14.8±1.3	16.4±1.3	20.7±2.2	23.4±1.9	**	NS	NS
**Leaf biomass (g DW**)	20	4.6±0.3	4.8±0.2	6.3±0.4	6.2±0.3	**	NS	NS
**Stem biomass (g DW**)	20	8.9±0.6	8.5±0.5	10.1±0.8	9.7±0.6	NS	NS	NS
**Root biomass (g DW**)	8	3.9±0.4	4.0±0.4	4.9±0.6	5.7±0.5	**	NS	NS
**Number of leaves**	20	42.8±3.5	48.1±4.5	61.0±3.8	55.3±3.3	**	NS	NS
**Number of branches**	20	6±0.6	8±0.8	9±0.7	8±0.7	*	NS	NS
**Total leaf area (cm** ^**2**^)	20	432±37	531±66	697±57	615±49	**	NS	NS
**Number of fallen leaves** ^***a***^	12	3.3 ± 0.7	3.0 ± 0.8	1.9 ± 0.7	1.6 ± 0.5	*	NS	NS
**Single leaf area (cm** ^**2**^)	20	11.4±0.6	11.7±0.6	12.5±0.7	12.3±0.5	NS	NS	NS
**SLA (cm** ^**2**^ **g DW** ^**−1**^)	20	152.8±2.8	160.7±4.7	153.3±4.7	154.7±4.0	NS	NS	NS
**Leaf water content (%**)	18–20	60.1±0.8	61.6±0.9	60.6±1.1	60.1±0.6	NS	NS	NS
**Stomatal density (mm** ^**−2**^)	10	184.0±7.8	195.1±10.9	184.3±8.1	167.9±5.7	NS	NS	NS
**Leaf vein density (mm mm** ^**−2**^)	10	7.0±0.2	7.0±0.2	7.0±0.2	6.6±0.3	NS	NS	NS
**Total N content per plant (g**)	8	0.20±0.02	0.21±0.02	0.22±0.02	0.23±0.02	NS	NS	NS

Statistically significant main effects for VPD and N treatments and their interaction were tested by two-way ANOVA (**P<0.01, *P<0.05). Values are means ±SE.

^*a*^ Number of fallen leaves between day 4 and day 20, calculated from photographs.

Plants produced 6–10 new long shoot leaves during the experiment (data not shown). The biomass of the new long shoot grown during the experiment was not affected by VPD or N treatments (data not shown). However, total biomass, root biomass, and leaf biomass were higher in low VPD plants than in high VPD plants (Table 1). Plants had more leaves and branches, and fewer fallen leaves under low VPD than under high VPD (Table 1). The numbers of leaves and branches were positively correlated (Pearson *r*=0.744, *P*<0.001). The mass fractions of leaves, stem, and roots were not significantly affected by VPD or N treatments (data not shown).

There was no difference in single leaf area, but the total leaf area was larger, since there were more leaves in low VPD plants than in high VPD plants (Table 1). There was no significant treatment effect on SLA, leaf water content, stomatal density, or leaf vein density (Table 1). Neither VPD nor N affected the total number of stomata, or vein length per leaf (data not shown).

Stomatal conductance of old leaves was higher in low VPD plants than in high VPD plants (Fig. 1B). Stomatal conductance showed a decreasing trend under low VPD, while it showed an increasing trend in high VPD treatment (Fig. 1B); thus, the difference between treatments diminished in time. N treatments did not affect stomatal conductance (Fig. 1B). The stomatal conductance values were low because the measured leaves were developed before the treatments started, leaves were large, and their stomatal density was low (data not shown).

### Chlorophyll, mineral nutrients, non-structural carbohydrates, and the expression of nitrogen-dependent genes

**Fig. 2.**
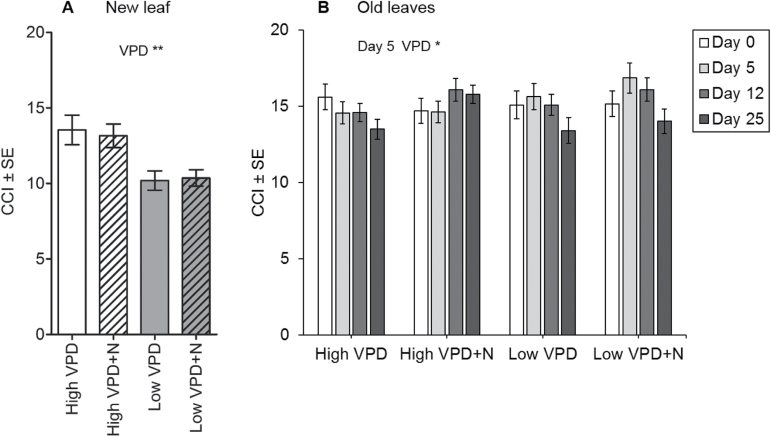
Chlorophyll content of new leaves (A) and old leaves (B) as affected by VPD and N treatments. Chlorophyll content of new leaves was measured at day 26 from one new fully expanded leaf that developed during the experiment (A). The chlorophyll content of old leaves (average of two leaves) that had developed before the experiment was measured at day 0, 5, 12, and 25 (B). Data are presented as mean ±SE, *n*=20; two-way ANOVA **P<0.01, *P<0.05.

The chlorophyll content of new leaves grown during the experiment was lower in plants that had grown under low VPD than under high VPD (Fig. 2A). The chlorophyll content of old leaves showed a transient increase under low VPD (at day 5), but later the chlorophyll content was at the same level as in high VPD samples with moderate N supply (Fig. 2B). Additional N supply increased the chlorophyll content of old leaves in high VPD plants and sustained it at a level ~17% higher than the moderate N supply (Fig. 2B).

**Fig. 3.**
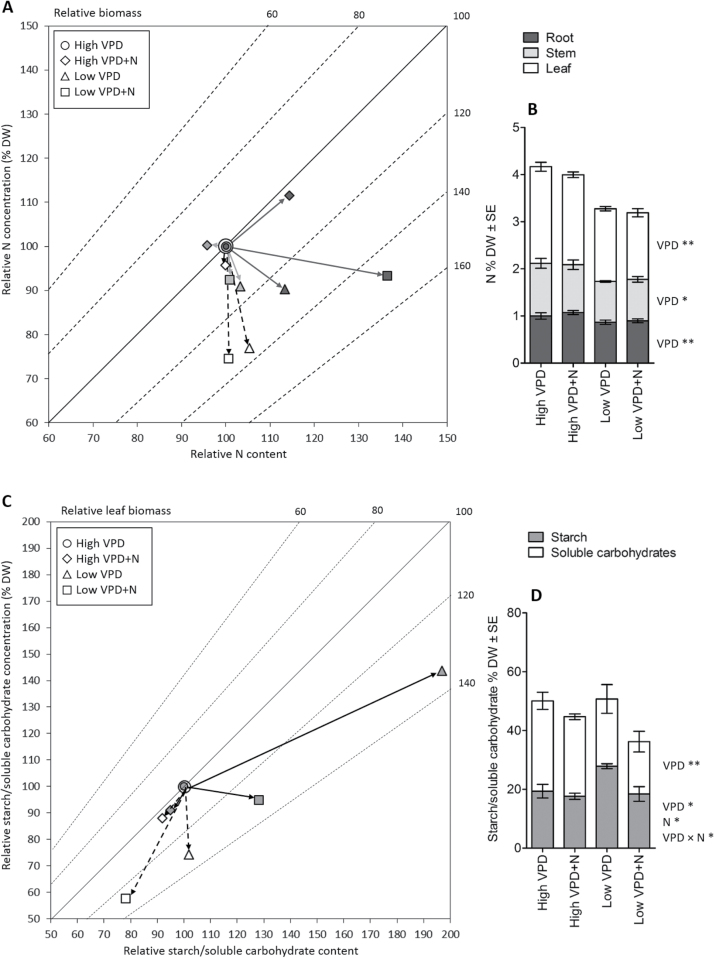
Graphical vector analysis (GVA) for the effects of VPD and N supply on the amounts of nitrogen (A, B) and carbohydrates (C, D). GVA displays changes in relative concentrations and absolute contents of nitrogen (A, B) and non-structural carbohydrates (starch and soluble carbohydrates) (C, D) in relation to biomass accumulation (day 26). GVA is constructed relative to high VPD treatment. Data are presented as mean ±SE, *n*=6–8 for nitrogen N % DW, *n*=5–6 for starch % DW, and *n*=20 for soluble carbohydrates % DW; two-way ANOVA **P<0.01, *P<0.05.

The estimated total N content of plants did not differ significantly between the VPD or N treatments (Table 1). The GVA showed that low VPD treatment affected N allocation between leaves, stem, and roots (Fig. 3A). N concentration (% DW) was significantly lower in new leaves, stem, and roots of low VPD plants than of high VPD plants (Fig. 3B), but the relative difference in N concentration was strongest in leaves (Fig. 3A). There was more N in the plant roots in high VPD treatment with high N supply than in high VPD with moderate N supply (Fig. 3A). In low VPD, the N concentration in root seemed to be diluted by root biomass (Fig. 3A).

The starch level was high in concentration and in absolute content in low VPD treatment with moderate N supply (Fig. 3C), which suggests excess production of starch in the leaves. Soluble sugars on the other hand were low in concentration in low VPD treatments and also in absolute content in low VPD treatment with high N supply (Fig. 3C). The total concentration of non-structural carbohydrates (NSCs) in leaves (sum of starch and soluble carbohydrates) did not differ significantly between the treatments (Fig. 3D). RHG % had a positive correlation with starch content of leaves (Pearson *r*=0.652, *P*=0.008).

**Fig. 4.**
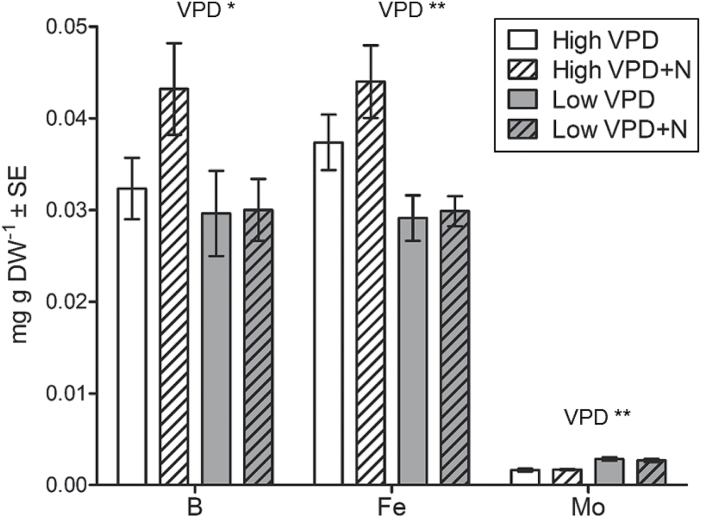
Concentration of boron (B), iron (Fe), and molybdenum (Mo) in new leaves that developed during the experiment. The leaves were sampled at day 26. Data are presented as mean ±SE, *n*=6; two-way ANOVA **P<0.01, *P<0.05.

In leaves formed under low VPD, the concentrations of boron (B) and iron (Fe) were lower and that of molybdenum (Mo) higher than under high VPD (Fig. 4). Concentrations of other mineral nutrients and the total nutrient concentration of new leaves were not significantly affected by VPD or N treatments (Supplementary Fig. S1).

**Fig. 5.**
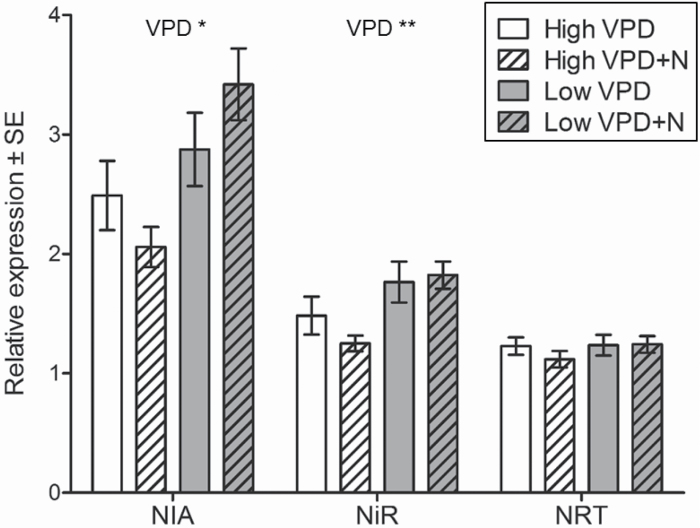
Relative expression of nitrate reductase (*BpNIA*), nitrite reductase (*BpNiR*), and nitrate transporter (*BpNRT*) in new leaves that developed during the experiment. The leaves were sampled at day 26. Elongation factor 1α (EF-1α) was used as a reference gene. Data are presented as mean ±SE, *n*=8; two-way ANOVA **P<0.01, *P<0.05.

The relative gene expression of nitrate reductase (*BpNIA*) and nitrite reductase (*BpNiR*) was higher in the leaves developed under low than under high VPD, but the expression levels did not show a significant response to N supply (Fig. 5). The expression of nitrate transporter (*BpNRT*) responded neither to VPD nor to N treatment (Fig. 5).

### Metabolite responses

**Fig. 6.**
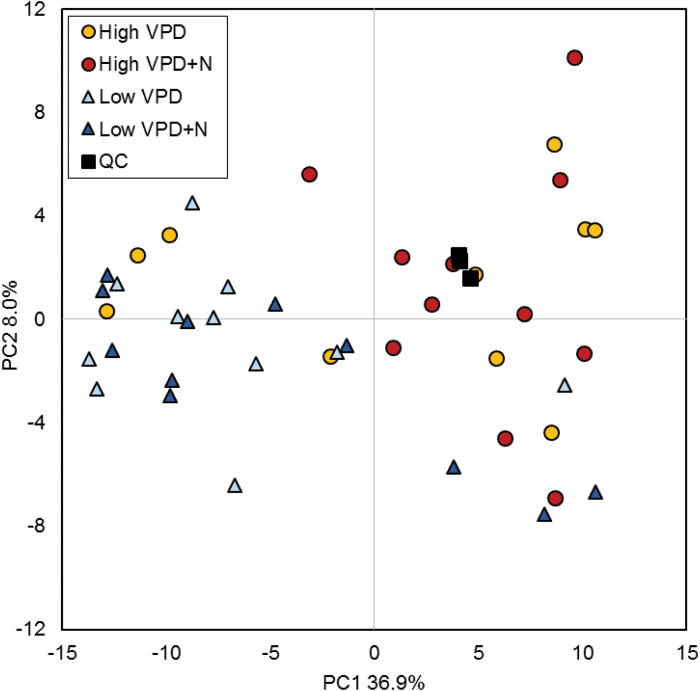
Principal component analysis (PCA) of metabolite data. PCA scores plot of metabolite samples (169 GC-MS metabolites+starch) includes quality control samples (QC) from three GC-MS runs. QC samples from three sample batches are grouped together. Most of the low VPD and high VPD samples are separated by the first principal component. *n*=10–11. (This figure is available in colour at *JXB* online.)

**Fig. 7.**
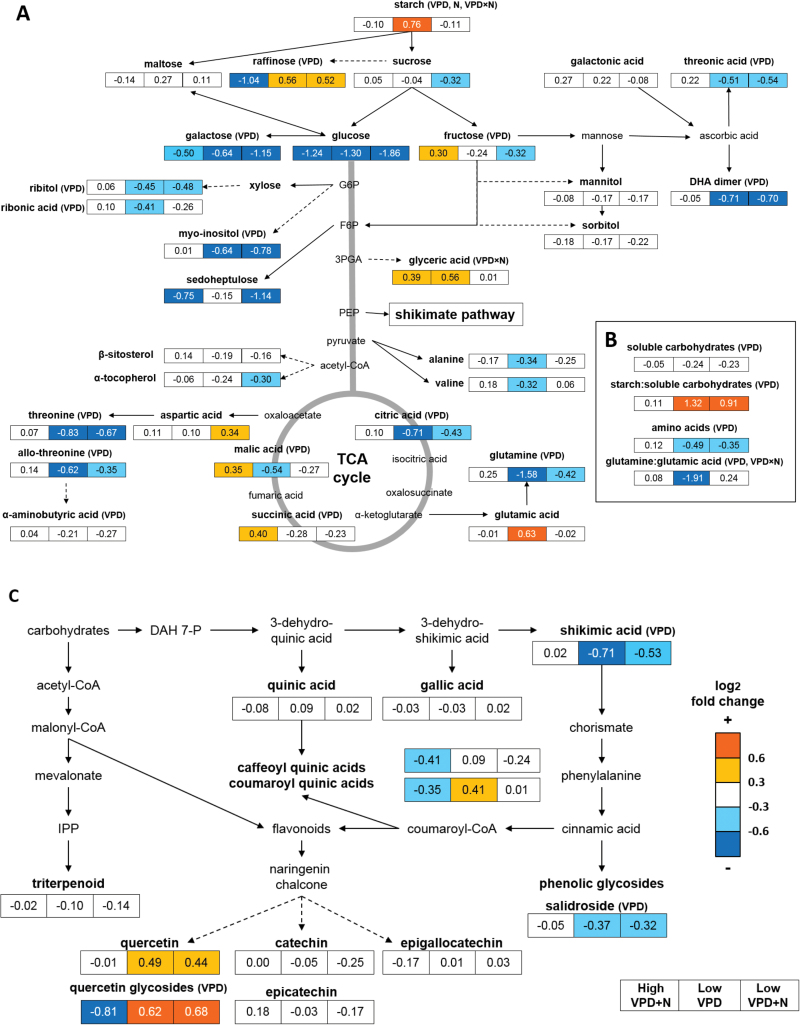
Overall scheme of the effects of VPD and N availability on the primary (A, B) and secondary metabolism (C) of silver birch leaves. New leaves that had developed during the experiment were sampled at day 26. Responses to high VPD + N (left), low VPD (middle), and low VPD + N (right) are presented as fold changes (log_2_) in the metabolite means compared with the high VPD plants. Significant treatment effects for VPD, N, and their interaction (VPD×N) are presented in parentheses next to the metabolite names/metabolite groups/metabolite ratios (two-way ANOVA *P*<0.05; FDR *q*<0.05 for individual GC-MS metabolites). *n*=20 for soluble carbohydrates, *n*=5–6 for starch; for GC-MS metabolites, see details in Supplementary Table S1. (This figure is available in colour at *JXB* online.)

PCA of the metabolite data (169 GC-MS metabolites and starch) separated most of the low and high VPD samples on the basis of the first component, explaining 36.9% of the total variation (Fig. 6). None of the first five principal components was related to N treatment. There was a strong metabolic response to low VPD; levels of four GC-MS metabolites (raffinose, *trans*-3-coumaroyl quinic acid, and two quercetin glycosides) were higher and the levels of 65 metabolites were lower in the leaves developed under low VPD (Fig. 7; Supplementary Table S1). The levels of sugars (fructose, gentiobiose, and ribonic acid), organic acids (citric acid, succinic acid, malic acid, and shikimic acid), amino acids (α-aminobutyric acid, threonine, allo-threonine, and glutamine), myo-inositol, salidroside (phenolic glycoside), and several unidentified compounds were lower in low VPD than in high VPD samples (Fig. 7; Supplementary Table S1).

The TCA cycle was affected by N supply under high VPD, demonstrated by higher levels of citric acid, succinic acid, and malic acid in the high VPD+N samples than in high VPD samples (Fig. 7A). Leaves that were developed in high VPD+N treatment contained lower levels of raffinose, *trans*-3-coumaroyl quinic acid, and quercetin glycosides than leaves that had grown in high VPD or in low VPD treatments (Fig. 7A, C). Overall, additional N supply under low VPD (low VPD+N) diminished the effect of low VPD treatment on the metabolite profile (Fig. 7; Supplementary Table S1A). Metabolic shift was therefore most pronounced between high VPD+N and low VPD samples (Figs 6, 7; Supplementary Table S1). An interaction effect of VPD and N was observed in the levels of glyceric acid and starch (Fig. 7A; Supplementary Table S1). The starch content of leaves was high only in low VPD samples with moderate N supply (Fig. 7A). However, the starch to soluble sugar ratio remained high under low VPD treatment regardless of the N treatment (Fig. 7B), because low VPD treatment reduced the total soluble carbohydrate content of the leaves (Fig. 7A, B).

Although individual amino acids responded differently to the treatments, the amino acid content (sum of alanine, valine, glutamate, glutamine, aspartate, threonine, allo-threonine, and α-aminobutyric acid) decreased with low VPD (Fig. 7B). Glutamate and aspartate levels were slightly higher, whereas the levels of other detected amino acids were lower in the low VPD samples than in the high VPD samples (Fig. 7B). The glutamine to glutamate ratio (Gln:Glu) was low in low VPD treatment, but with additional N supply, in low VPD+N, the ratio was at the same level as in high VPD samples (Fig. 7B).

## Discussion

### High air humidity modifies the growth of silver birch

Biomass accumulation of birch plants was promoted by low VPD, which is a common response of plants to high air humidity (Schüssler *et al.*, 1992; Mortensen, 2000; Leuschner, 2002). However, the stem elongation rate and stomatal conductance of old leaves showed transient acclimation regimes. Due to the opposite trends in low and high VPD plants for height growth rate and stomatal conductance, the large differences observed at the beginning of the experiment decreased over time. A longer experiment would be needed to see if the growth rate and stomatal conductance would level out, or if the response curves would intercept eventually in the two VPD conditions. A transient increase in growth rate has been observed in tomato plants (Armstrong and Kirby, 1979) and aspen trees (Tullus *et al.*, 2012) under low VPD. The growth of hybrid aspen was enhanced during the first growing season under low VPD in the FAHM field, but the growth rate slowed down during the second season of humidity manipulation (Tullus *et al.*, 2012). Cunningham (2006) reported that several temperate and tropical trees showed non-significant increases (<10%) in growth under low VPD after a year. Since low VPD promotes stomatal opening and reduces transpiration rate (Bunce, 1984; Fordham *et al.*, 2001), height growth of birch may have initially been enhanced in the low VPD treatment due to increased carbon fixation and ameliorated water status.

Although low VPD may first lead to enhanced growth, the growth rate may eventually slow down due to impaired nutrition and altered resource allocation patterns. Root and leaf biomass production and radial stem growth were promoted under low VPD, while additional N supply under low VPD mostly promoted root growth. High humidity enhanced the fine root biomass and the formation of root tips in birch trees in the FAHM field study (Parts *et al.*, 2013; Rosenvald *et al.*, 2014). Altered root morphology and increase in root biomass are typical responses to low nutrient availability (de Groot *et al.*, 2003; Chapman *et al.*, 2012; Luo *et al.*, 2013). Similarly to our observation, the HDR of birches was lower in elevated humidity than in ambient humidity in the FAHM field study (Sellin *et al.*, 2013). However, in our study, stem diameter increased more than height, whereas, in the field, stem diameter growth was less inhibited than height growth under high humidity (Sellin *et al.*, 2013).

Although an increase in single leaf area and SLA is a common response to low VPD (Choi *et al.*, 1997; Codarin *et al.*, 2006; Lendzion and Leuschner, 2008; Carins Murphy *et al.*, 2013), we did not observe significant changes in the single leaf area or SLA of new leaves developed during the experiment. As low leaf N has been associated with low turgor and a low expansion rate of leaves (Radin and Boyer, 1982), here low N concentration may have hindered leaf expansion under low VPD. However, birches grown under low VPD had more leaves and thus more transpiring and photosynthesizing leaf area than those grown under high VPD. The number of leaves has been observed to increase with low VPD in beech (Lendzion and Leuschner, 2008) and roses (Mortensen *et al.*, 2001). Our study revealed that the increase in the number of leaves in silver birch resulted from induced branch formation together with few fallen leaves in low VPD. In line with these results, silver birches grown under elevated air humidity in the FAHM field experiment had more branches (Sellin *et al.*, 2013) and longer foliage life span (Godbold *et al.*, 2014) than did control trees. Often a long life span of leaves is associated with efficient conservation of nutrients (Escudero *et al.*, 1992; Eckstein *et al.*, 1999). In this study, the lower number of fallen leaves and the changes in the chlorophyll content of old leaves suggest that N was efficiently recycled in the plants under low VPD. Large total leaf area together with reallocation of N, enhanced root growth, and radial stem growth could compensate for reduced transpiration rate, water transport, and nutrient acquisition, which are typically restricted by low VPD.

### High air humidity affects nitrogen metabolism

As expected, N concentrations of plant tissues were lower in low VPD than in high VPD. GVA showed that the change in N concentration in response to VPD and N treatments differed between leaves, stem, and roots; the N concentration of leaves showed the strongest decline in response to low VPD. According to Leuschner (2002), the decrease in nutrient concentration under low VPD may result from a dilution effect caused by increased carbon assimilation. However, total NSC concentration was not higher, and thus it did not account for dilution of N in the leaves exposed to low VPD. Only N and Fe concentrations were significantly lower in the new leaves in low than in high VPD, which does not support a dilution effect. This implies that there are additional processes contributing to low N concentration in the leaves in low VPD.

A positive relationship between transpiration rate and N uptake has been previously observed with tropical trees (Cernusak *et al.*, 2009) and northern trees (McDonald *et al.*, 2002; Kupper *et al.*, 2011; Tullus *et al.*, 2012). Similarly, a positive relationship between transpiration rate and Fe uptake and partitioning to leaves has been reported in rice (Shrestha *et al.*, 2015). Besides N, Mg and Ca are also delivered to the roots through the mass flow of water, and their concentrations have been reported to decline in low VPD (Armstrong and Kirby, 1979; Choi *et al.*, 1997) regardless of nutrient supply (Suzuki *et al.*, 2015). Here, total N content per plant and Mg and Ca concentrations of leaves were not affected by VPD, which does not support the hypothesis of reduced nutrient uptake by transpiration-driven mass flow of water in low VPD. In this study, higher root biomass in low than in high VPD and relatively small pot size may have facilitated root contact with nutrients and nutrient acquisition despite the reduced transpiration rate. Low VPD affects nutrient distribution in plants, mainly reducing the nutrient concentration of young leaves (Armstrong and Kirby, 1979; Choi *et al.*, 1997; Shrestha *et al.*, 2015), which was also supported by our results. Thereby changes in the allocation patterns appear to contribute to the low N and Fe concentrations of new leaves in low VPD.

Apart from N, Fe is also an essential element in chlorophyll biosynthesis; it is a cofactor for the glutamyl-tRNA reductase enzyme needed for chlorophyll production from 5-aminolaevulinic acid (Tanaka and Tanaka 2007). A decline in chlorophyll content indicated that less N was allocated to the photosynthetic machinery in low VPD. Fe and Mo are required as cofactors for the N assimilatory enzymes, nitrate reductase (NiA), nitrite reductase (NiR), and glutamate synthetase (GOGAT) (Mendel and Hänsch, 2002; Bittner, 2014). There is a tight link between N, Fe, and Mo metabolism, and all those elements showed significant changes in response to low VPD.

In contrast to our hypothesis, the leaf mRNA levels of nitrate reductase (*BpNIA*) and nitrite reductase (*BpNiR*) were higher, although the N concentration of the leaves was lower in low VPD than in high VPD. Typically, NIA and NiR transcription and enzyme activities are induced by nitrate and N supply (Crawford and Glass, 1998). Apart from N, light, carbohydrates, N metabolites, and the circadian clock are also involved in the regulation of N-related gene expression (Stitt *et al.*, 2002). There are three possible hypotheses for the unexpected gene expression patterns observed under low VPD. High Mo content of leaves may induce NIA transcription, as increased Mo content was found to induce NIA activity in sugar beet and pea plants (Kevresan *et al.*, 1998, 2001). However, in excess levels, Mo inhibits NIA activity (Srivastava, 1980). Alternatively, high mRNA levels under low VPD could be due to feedback inhibition of gene expression in high VPD, since the glutamine level was high under high VPD, and feedback regulation of N-related genes is mainly derived by glutamine (Gojon *et al.*, 1998). This hypothesis is supported by slightly lower mRNA levels in high VPD+N samples than in high VPD samples. Thirdly, high mRNA levels under low VPD may not reflect the actual enzyme activities, since NIA and NiR transcript levels can accumulate even despite low enzyme activity, as shown by the chemical inhibition of NIA (Migge *et al.*, 1997) and in *nia* mutants with an inoperative/deficient enzyme (Faure *et al.*, 1991; Vaucheret *et al.*, 1992).

The glutamine and glutamate contents of leaves are affected by the rate of nitrate reduction and photorespiration. Since the high ratio of glutamine to glutamate indicates high ammonium assimilation and high activity of the GS/GOGAT pathway (Foyer *et al.*, 2003), the ratio of glutamine to glutamate was used to estimate the N assimilation capacity of plants. Here, the low Gln:Glu ratio in leaves under low VPD indicated a low ammonium assimilation rate. High Mo fertilization and high Mo concentration of leaves have been shown to decrease GS activity in pea plants (Kevresan *et al.*, 2001), but the opposite response has been observed in sugar beet (Kevresan *et al.*, 1998). The process behind increased Mo in response to low VPD remains to be verified, but it may be linked to regulation of N metabolism in response to N limitation.

Metabolite responses to low VPD resembled symptoms of poor N status more than Fe deficiency. Fe limitation has been shown to increase the levels of organic acids and amino acids (Rellan-Alvarez *et al*., 2011; Borlotti *et al.*, 2012 *), whereas N limitation has been shown to decrease the levels of organic acids and amino acids, glutamine in particular* (Geiger *et al.*, 1999; Scheible *et al.*, 2004; Howarth *et al.*, 2008). In contrast to other amino acids, glutamate and aspartate levels tended to be higher under low than under high VPD, which has also been observed in low N conditions in tobacco leaves (Fritz *et al.*, 2006*b*). The decline in chlorophyll content, amino acids, TCA cycle intermediates, and the Gln:Glu ratio reflected N limitation in low VPD conditions.

### High air humidity affects carbohydrate metabolism

Since N and carbon metabolisms are tightly integrated processes, there was a close relationship between N, chlorophyll, and soluble carbohydrates—all declined under low VPD. N limitation inhibits photosynthesis, which is presumably the reason why the photosynthetic capacity of birch trees decreased under elevated humidity in the FAHM field (Sellin *et al.*, 2013). The growth of sink tissues is typically more restrained under stress conditions than is photosynthesis, thereby leading to the accumulation of photosynthates in the leaves. The low N status and amino acid content of leaves in low VPD appeared to restrict utilization and export of sucrose and induced carbon storage as starch, which is often observed in N-limited plants (Paul and Driscoll, 1997; Scheible *et al.*, 1997). Although additional N supply under low VPD restricted excessive starch production, the starch content remained high relative to soluble carbohydrates. A similar response has been observed in tobacco leaves in response to low N conditions, especially under elevated atmospheric CO_2_ (Geiger *et al.*, 1999). The decreasing trend observed for stomatal conductance in low VPD may reduce excess CO_2_ influx to the mesophyll, consequently adjusting the photosynthetic rate according to N assimilation rate.

Carbon storage as poly- and oligosaccharides was further indicated by the increased raffinose content under low VPD regardless of the N treatment. High levels of raffinose group sugars have been observed under N limitation in maize leaves (Schlüter *et al.*, 2012, 2013). The higher levels of starch and raffinose in the leaves developed under low VPD than under high VPD may relate to altered sink–source relationships in plants. The main transportable carbohydrate in plants is sucrose, but some species also transport raffinose, stachyose, and sugar alcohols (Lalonde *et al.*, 2004). If sink strength decreases, phloem loading may be inhibited, leading to increased starch and raffinose content in the leaves (Lemoine *et al.*, 2013).

The production of carbon-based secondary metabolites through the phenylpropanoid pathway is a common response to nutrient deficiency (Koricheva *et al.*, 1998; Amtmann and Armengaud, 2009; Schlüter *et al.*, 2013) and low leaf nitrate content (Fritz *et al.*, 2006*a*). The production of flavonoids was promoted by low VPD and accompanied by low leaf N content in silver birch.In contrast, the level of shikimic acid, which is the precursor for phenylpropanoids, was lower in low VPD, indicating efficient biosynthesis of downstream products. The production of quinic acid derivatives was down-regulated by additional N supply, especially under high VPD. Flavonoid levels, on the other hand, were higher in low than in high VPD regardless of N supply. Previous studies with silver birch have shown that flavonols and flavonol glycosides increase with N deficiency (de la Rosa *et al.*, 2001; Keski-Saari *et al.*, 2005). Similarly, transcript and metabolic studies with Arabidopsis have shown that N deficiency promotes the production of flavonoids and induces glycosyl transferase genes involved in glycosylating flavonoids (Scheible *et al.*, 2004).

### Conclusion

In this short-term growth chamber experiment, growth, biomass accumulation, nutrient status, leaf metabolism, and N-dependent gene expression patterns were strongly affected by VPD treatment. The effects of N supply and VPD×N interaction were mainly observed at the metabolic level. It is possible that the short time scale, relatively small pot size, and initial N content in the substrate may have attenuated the N treatments. Metabolic adjustments and mineral nutrient responses to low VPD indicated that N assimilation did not keep up with the carbon assimilation rate. Low levels of N, Fe, chlorophyll, amino acids, and soluble carbohydrates, and high levels of starch, raffinose, and flavonoids provided evidence of N limitation in low VPD. Additional N supply diminished the effects of low VPD on the metabolite profile, suggesting that metabolic responses to low VPD were mainly driven by N availability. This study shows that VPD is a highly important factor that affects the carbon and nutrient metabolism of plants and plant responses to N supply.

## Supplementary data

Supplementary data are available at *JXB* online.

Table S1. Leaf metabolites, annotation details, and metabolite responses to VPD and N treatments.

Table S2. Primers used in real-time quantitative PCR.

Figure S1. Mineral nutrient concentrations in new leaves as affected by VPD and N treatments.

Supplementary Data

## Abbreviations:

FAHM: free air humidity manipulation
FDR: false discovery rate
GOGAT: glutamate synthase
GS: glutamine synthetase
GVA: graphical vector analysis
HDR: height to diameter ratio
LWC: leaf water content
NIA: nitrate reductase
NiR: nitrite reductase
NRT: nitrate transporter
NSC: non-structural carbohydrate
QC: quality control
RDG: relative diameter growth
RH: relative humidity
RHG: relative height growth
SLA: specific leaf area
TCA: tricarboxylic acid
VPD: vapour pressure deficit.
