# Supplementary material for: Low vapour pressure deficit affects nitrogen nutrition and foliar metabolites in silver birch
Source: J Exp Bot. 2016 Jun 3;67(14):4353–65. doi: 10.1093/jxb/erw218 (PMC5301935; doi:10.1093/jxb/erw218)
Supplement: Supplementary Data [file supp_67_14_4353__index.html]

Low vapour pressure deficit affects nitrogen nutrition and foliar metabolites of silver birch — Low vapour pressure deficit affects nitrogen nutrition and foliar metabolites in silver birch — Supplementary Data 

# Low vapour pressure deficit affects nitrogen nutrition and foliar metabolites in silver birch

## Supplementary Data

Data files

- Supplementary\_table\_S1.xlsx - Supplementary Data
- supplementary\_table\_S2\_figure\_S1.pdf - Supplementary Data
