# Supplementary material for: Low vapour pressure deficit affects nitrogen nutrition and foliar metabolites in silver birch
Source: J Exp Bot. 2016 Jun 3;67(14):4353–65. doi: 10.1093/jxb/erw218 (PMC5301935; doi:10.1093/jxb/erw218)
Supplement: Supplementary Data [file supp_erw218_supplementary_table_S2_figure_S1.pdf]

Supplementary Table S2.

Primers used in quantitative real-time PCR. Tubulin primers are described in Ibrahim et al. (2010).

**Reference genes**	**Sequence**	**amplicon length (bp)**
actin<br>*(B. pendula EST)*	F: GGATGGAAGCTGCTGGAATAC<br>R: GTTGGAAGGTGCTGAGAGAAG	258
Ubiquitin<br>(*B. pendula* EST)	F: AGGTGGAGAGCTCCGATACC<br>R: GATTGTGTCCGAGCTCTCAAC	251
Elongation factor 1α<br>(*B. pendula* EST)	F: TCGACCACCACTGGTCATTTG<br>R: GCCTGTGAGGTACCGGTAATC	266
α-Tubulin<br>(AJ279695)	F: CGGTTCGATGGAGCCTTGAA<br>R: CATCAAGCAGCAAGCCATGT	228
**Target genes**		
Nia1<br>Nitrate reductase	F: CTCCAACGAGCACCATTGATG<br>R: GCTAGCCTCTTGGCAAACTTG	226
NiR<br>Nitrite reductase	F: ACTGGACAGGCTGTCCAAATAG<br>R: CCCTTGGTACAGCTCCAAAATG	237
NRT<br>Nitrate transporter	F: GCTGTTCTCATCACCACCATTC<br>R: TCCAGAACCCACTAGGAAGAAC	255

Actin EST= *B. pendula* EST with a 99% identity to *B. platyphylla* actin (GenBank Acc HO112155)
Ubiquitin EST= *B. platyphylla* ubiquitin (HO112156)
Elongation factor 1α = Q9XEW9

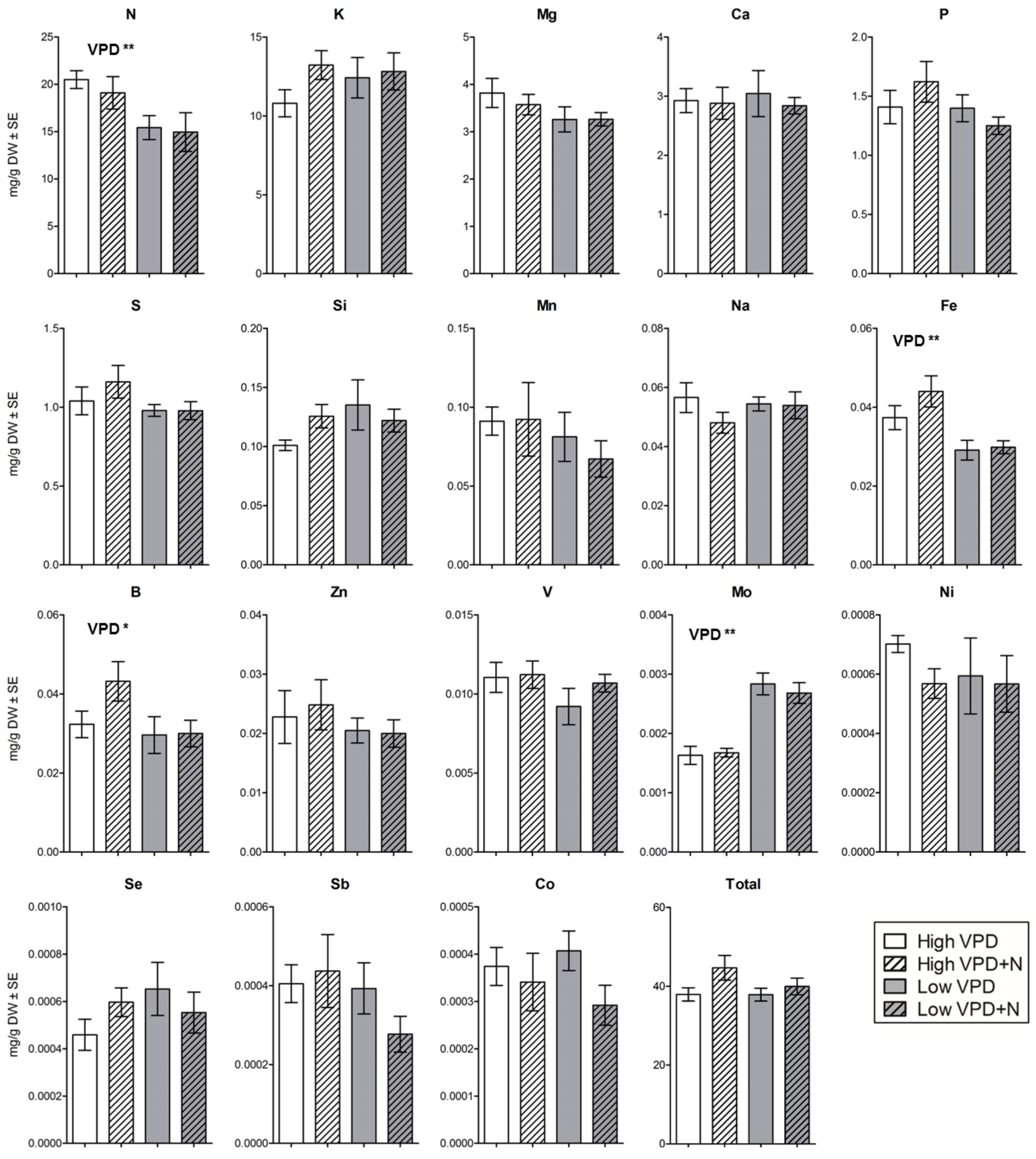

Supplementary Figure S1. Mineral nutrient concentrations in new leaves as affected by VPD and N treatments. The leaves were harvested at day 26. Note different scales on x-axis. Data represented as mean ± SE. n=3-6, two-way ANOVA ** $p<0.01$, *$p<0.05$
